# A Ka-band one-dimensional beam scanning leaky-wave antenna based on liquid crystal

**DOI:** 10.1038/s41598-024-54688-4

**Published:** 2024-02-16

**Authors:** Shunhu Hou, Shengliang Fang, Yangyang Wang, Mengtao Wang, Yuxin Wang, Jinlong Tian, Junhao Feng

**Affiliations:** 1 Graduate School, Space Engineering University, Beijing, 101416 China; 2 School of Space Information, Space Engineering University, Beijing, 101416 China; 3 Graduate School, Rocket Force University of Engineering, Xian, 710025 China; 4 Graduate School, National University of Defense Technology, Changsha, 410073 China

**Keywords:** Metamaterials, Sub-wavelength optics, Aerospace engineering, Electrical and electronic engineering

## Abstract

Fixed frequency beam-scanning leaky-wave antennas have been a focus of attention for many scholars in recent years, and numerous related results have been obtained. However, these antennas suffer from several issues such as small beam-scanning range, low gain, and unsatisfactory impedance matching. To address these problems, this paper proposes a microstrip line (ML) antenna unit based on liquid crystal (LC) materials etched Complementary Split Ring Resonator (CSRR). In a first-of-its-kind approach, the substrate integrated waveguide (SIW) structure and the ML transmission structure are combined to present the SIW-ML transmission structure. The antenna operates in the Ka-band with excellent resonance characteristics at 34.7 GHz, and the S11 parameters are below − 13 dB in the frequency range of 30–40 GHz, indicating outstanding impedance matching. By arranging 56 antenna units, a periodic leaky-wave antenna is created, enabling fixed-frequency beam-scanning at 34.7 GHz. Experimental results show that the antenna can achieve scanning of angles between − 53° and + 60° with a gain of up to 12.63 dB. Once single-beam scanning is achieved, a method combining LC and discrete amplitude weighting technique, as well as multi-beam theory, is proposed for multi-beam study. Experimental results reveal that the designed 56-unit beam-scanning antenna can effectively realize beam scanning in two directions.

## Introduction

Antennas, as devices that transmit and receive electromagnetic waves, play a crucial role in wireless communication systems. They find wide applications in various fields such as radio communication, navigation, radar, electronic countermeasures, and serve essential functions. Different application areas have diverse requirements for antenna performance, and among them, the beam-scanning capability of an antenna is vital for achieving system functionalities. Since the introduction of leaky-wave antennas in the 1940s, they have attracted considerable attention from scholars due to their unique radiation characteristics and excellent beam-scanning abilities. In recent years, research on leaky-wave antennas has witnessed significant growth, resulting in the emergence of various forms of such antennas^1–5^. Leaky-wave antennas offer not only high efficiency but also advantages like low profile, compact size, and simple feeding structure^6–9^. However, traditional leaky-wave antennas are designed for frequency beam scanning, which limits their utility in today's environment where spectrum resources are scarce. To tackle this challenge, it becomes imperative to explore leaky-wave antennas with fixed-frequency beam scanning capabilities^10–14^.

To achieve control of the antenna beam, researchers are increasingly relying on the use of tunable materials. Common tunable materials include ferroelectric materials, optoelectronic materials, temperature-varying materials, and LC materials. These materials serve as the transmission medium of the leaky-wave antenna, and by adjusting their characteristics, the direction and range of the antenna beam can be controlled. Currently, LC materials are gaining popularity, as they can be used in millimeter wave and higher frequency bands, and are increasingly being combined with leaky-wave antennas to achieve fixed-frequency beam scanning^15–21^. However, most of the studies have problems such as low frequency band, small beam scanning range or low gain^22–27^. Wide beam scanning range, high gain, and high-frequency bands are crucial evaluation criteria in leaky-wave antennas. These factors also pose design challenges, making them the focal point of attention among researchers. In 2020, Qi Liu designed a 64-cell beam-scanning microstrip line leaky-wave antenna utilizing LC technology^28^. This antenna operates at 30 GHz and enables beam scanning from − 27° to + 38°, with a gain range of 9.5–12.5 dB. Elahehsadat Torabi proposed a wide-beam substrate integrated waveguide leaky-wave antenna based on LC^29^. Operating at 13.6 GHz, this antenna achieves a beam scanning range of 56° and boasts the unique advantage of high gain and low sidelobes. Francesca Imperato proposed a unidirectional leaky-wave antenna based on nematic liquid crystal (NLC)^30^. Operating at 1.0864 THz, it offers a wave velocity scanning range of 20° while maintaining an almost constant 10° beamwidth. Henning Tessmer proposed a reconfigurable LC dielectric image line leaky-wave antenna with a high gain of 18 dBi and a beam scanning range from − 30° to + 10°^31^.

In addition to the advantages of high gain and wide beam scanning range, the leaky-wave antenna also needs to be rationally designed structure to obtain excellent impedance matching and small size superiority. Yingxiang Jia proposed a parallel-plate waveguide leaky-wave antenna^32^. With a return loss of less than − 12 dB in the frequency band of 17.8–22.6 GHz, it offers a sweeping range of 96° at 22.6 GHz and a gain exceeding 9 dBi. Lin Zhu Wang proposed a continuously tunable frequency microstrip antenna based on LC^33^, suitable for the Ka-band. It exhibits a resonant frequency agility range of 27.9/31.42–28.04/30.34 GHz, with a return loss below − 20 dB. Henning Tessmer et al. presented an all-dielectric LC electronically controlled scanning antenna operating in the V-band (50 GHz)^34^. This antenna significantly reduces the antenna weight. Weiyi Zhang proposed an LC substrate integrated waveguide leaky-wave antenna based on holographic theory^35^. It achieves a beam scanning range of − 45° to + 51° at 35 GHz, with a reflection coefficient below − 13 dB. According to the current state of research, we observe that designing leaky-wave antennas with high frequency band, high gain, broad beam scanning range, low return loss, and compact size represents a challenging issue of great significance.

Based on recent research advancements in LC-based beam-scanning leaky-wave antennas, the progress of existing research findings is summarized in Table 1 below.

**Table 1:** Summary of research progress.

Attribute	Attribute interval division and the proportion of existing achievements		
Operating frequency	Below 30 GHz (77.5%)	Above 30 GHz (22.5%)	
Beam scanning range	Below 50° (47.2%)	50°–100° (38.9%)	Above 100° (13.9%)
Antenna gain	Below 7 dB (28.6%)	7–12 dB (46.4%)	Above 12 dB (25%)

Table 1 presents that the majority of current research on LC-based leaky-wave antennas focuses on frequencies below 30 GHz, with relatively fewer studies conducted for frequencies above 30 GHz. Only around 13.9% of the antennas achieve a beam scanning range of more than 100°, and approximately 25% achieve a beam gain of over 12 dB. This indicates that achieving large beam-scanning ranges and high beam gains are critical research areas, but also pose significant challenges. In light of the current issues such as limited beam-scanning range, low gain, unsatisfactory impedance matching, and low operating frequency in this field, this paper proposes a ML antenna unit based on LC materials etched CSRR. The main innovations are outlined below:

1. The introduction of an antenna structure based on the etched CSRR of LC, marking the first application of this technology in the field of leaky-wave antennas. Additionally, the SIW-ML transmission structure is proposed in a first-of-its-kind approach by combining the SIW structure with the ML transmission structure. The SIW-ML structure enhances energy coupling effects, improving signal transmission efficiency and reducing losses in waveguide transmission. Moreover, the presence of metal pillars helps minimize beam spread, allowing for more focused signal transmission and better impedance matching.
2. The antenna unit exhibits key advantages such as a wide frequency band, large angle scanning range, high gain, and excellent impedance matching performance. Specifically, the antenna enables angle scanning from − 53° to + 60° at 34.7 GHz, with a gain of 12.63 dB. Furthermore, the S11 parameter remains below − 13 dB throughout the 30–40 GHz range.
3. For the first time, the project presents a method that combines LC materials, binary discrete amplitude weighting technique, and multi-beam theory to achieve the multi-beam performance of a leaky-wave antenna. Experimental results demonstrate successful dual-beam scanning capability of the designed 56-unit antenna.

These innovations provide new ideas and lay the foundation for the further development of LC-based leaky-wave antennas.

## Design and methods

### Antenna structure design

**Figure 1:**
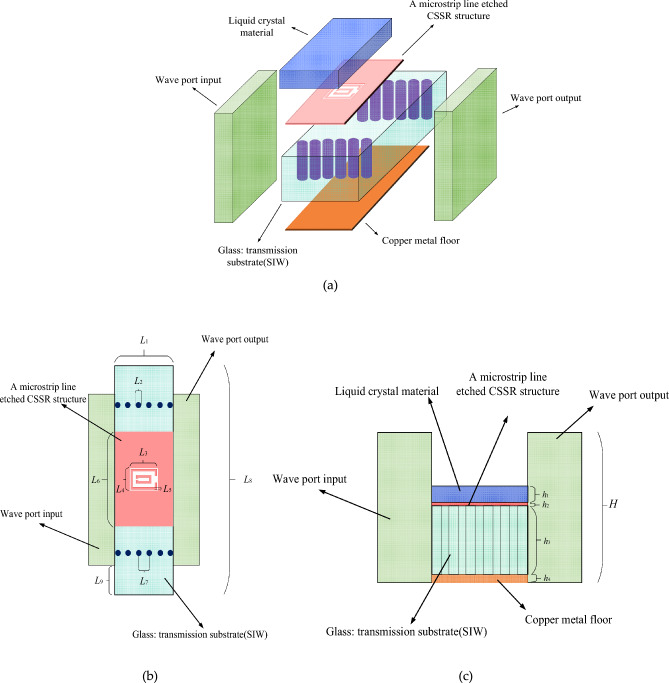
Schematic diagram of the structure of the antenna unit. (**a**) Main view; (**b**) top view; (**c**) front view.

Figure 1 illustrates the structure of the metamaterial unit incorporating LC material. The unit structure consists of a copper metal floor, a dielectric substrate, a microstrip line etched CSSR structure, and a LC layer in order from bottom to top. The dielectric substrate is a 1.5-mm-thick Glass sheet with a relative permittivity of 5.5. The copper plate on the bottom of the dielectric substrate serves as the grounding surface, and the thickness of the copper plate is 0.035 mm. The microstrip transmission line of the etched CSSR unit is made of copper metal with a thickness of 2 µm and is covered by a 0.2-mm-thick LC layer above it. Excitation is achieved through wave-port configurations, utilizing two ports: one for feed input and the other for output.

The antenna design employs GT3-23001 LC material. The dielectric constant of this type of LC varies from 2.5 to 3.3. This variation in dielectric constant leads to a shift in the resonant frequency of the resonator, subsequently impacting the energy coupled out from the slots and the radiation efficiency. We use HFSS electromagnetic simulation software for antenna structure design and performance simulation. This article mainly considers the influence of the dielectric constant of LC materials on the unit, so materials with the same dielectric constant are used as equivalent substitutes in the design.

As shown in Fig. 1, the dimensional parameters of this antenna unit are shown in Table 2.

**Table 2:** Antenna size parameters.

Variables	$L_{1}$	$L_{2}$	$L_{3}$	$L_{4}$	$L_{5}$
Size/mm	2	0.2	1.36	0.8	0.09

Variables	$L_{6}$	$L_{7}$	$L_{8}$	$L_{9}$	$h_{1}$
Size/mm	3	0.3	8.6	0.8	0.2

Variables	$h_{2}$	$h_{3}$	$h_{4}$	H	–
Size/mm	0.002	1.5	0.035	2.7	–

### Antenna unit equivalent circuit

**Figure 2:**
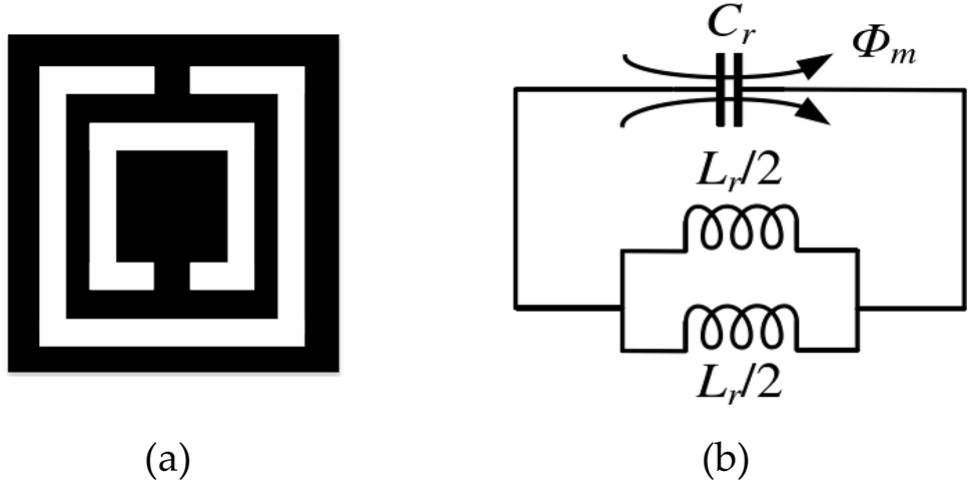
Structure of CSSR and its equivalent circuit.

The Complementary Split Ring Resonator (CSRR) is a complementary form of the Split Ring Resonator (SRR). SRR comprises two metal rings with identical centers and back-to-back openings and has negative magnetic permeability characteristics similar to those observed in CSRR. When excited by an axial magnetic field, the CSRR can be regarded as a magnetic dipole. At the same resonant frequency, the CSRR composed of multiple metal rings with the same center of the circle is compact compared to that composed of a single metal ring, rendering the device more miniaturized. Figure 2 illustrates both the structure of the CSRR and its equivalent circuit. In Fig. 2a, the total capacitance of the gap between the inner and outer rings is $C_{r}$, and the average side length and average width of the inner and outer rings of the CSRR are equivalent to two inductors $L_{r} /2$ connected in parallel. Hence, the resonant frequency of the CSSR structure can be expressed as:

1 $$f_{CSRR} = \frac{1}{{2\pi \sqrt {(C_{r} \cdot L_{r} /4)} }} = \frac{1}{{\pi \sqrt {C_{r} L_{r} } }}$$

In the whole structure of the leaky wave antenna unit, the microstrip line, the dielectric substrate and the metal ground plate form a resonant circuit, which can be equated to an RCL parallel circuit. Wherein, the inductance (L) is an inductive element constituted by the space between the microstrip line and the metal grounding plate, and its inductance value can be calculated by the formula $L = \mu_{0} \cdot \varepsilon_{{\text{r}}} \cdot l/w$. $\mu_{0}$ is the free-space magnetic permeability (about 4*π* × 10 H/m), $\varepsilon_{r}$ is the relative permittivity of the dielectric layer, l is the length of the microstrip line, and w is the width of the microstrip line. The capacitance (C) is a capacitive element formed by the dielectric layer between the microstrip line and the metal ground plate, and its capacitance value can be calculated by the formula $C = (\varepsilon_{0} \cdot \varepsilon_{r} \cdot A)/h$. $\varepsilon_{0}$ is the dielectric constant in vacuum (about 8.854 × 10–12 F/m), A is the effective area of the microstrip line, and h is the thickness of the dielectric layer. There are some losses in the microstrip line leaky antenna, which can be considered as an equivalent resistance R, which is related to the material as well as the geometrical parameters of the microstrip line. From the above calculation, the resonant frequency of the RCL parallel circuit can be calculated by the following equation: $f = 1/(2\pi \cdot sqrt(L \cdot C))$.

**Figure 3:**
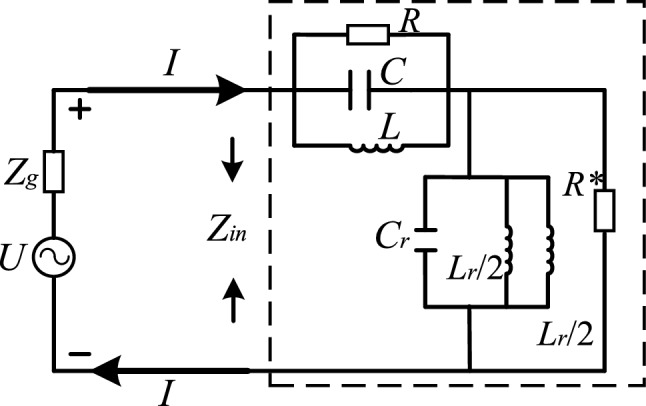
Equivalent circuit diagram of antenna structure.

By etching the CSRR structure on the microstrip line, another new resonant circuit is introduced, which is equivalent to connecting a CL parallel circuit in series with the original RCL parallel circuit. The equivalent circuit of the whole antenna structure is shown in Fig. 3 below. $R^{ * }$ is the output port load resistor, and the resistor resistance value is 50 ohms.

The antenna structure feed is a mode-driven wave-port excitation with an excitation energy of 1 W. The internal resistance of the power supply is set to 50 ohms (Ω). The output impedance of the power supply is denoted by $Z_{in}$ and expressed as follows ($W = 2\pi * f_{CSRR}$):

2 $$Z_{in} = \frac{U}{I} = \frac{jWLR}{{jWL + R - LW^{2} R^{2} }} + \frac{{jWL_{r} }}{{4 - W^{2} L_{r} C_{r} }}/R^{ * }$$

Impedance bandwidth is a crucial indicator of antenna performance, representing the bandwidth corresponding to the reflection coefficient of the antenna when S11 <  − 10 dB. When the output impedance of the antenna feed power is not matched with the input impedance of the load, the input electromagnetic wave will be partially reflected. A severe impedance mismatch will result in an excessive reflection coefficient S11, which is not technically desirable. Therefore, the impedance matching of the antenna is of utmost importance.

### Holographic antenna principle

To achieve beam scanning capability for the leaky-wave antenna, an amplitude weighting method is employed. The method is realized based on the holographic antenna principle^36,37^. The holographic antenna mainly consists of a source antenna and a holographic structure. The source antenna corresponds to the reference wave, while the radiated wave to be generated corresponds to the object wave. The holographic structure records the holographic pattern generated by the interference between the reference wave and the object wave. Taking the one-dimensional structure as an example, the reference wave and object wave expressions are expressed as Eqs. (3) and (4), respectively:

3 $$\psi_{ref} (x_{n} ) = \exp \left( { - jk_{r} x_{n} } \right)$$

4 $$\psi_{obj} (x_{n} ,\theta_{0} ) = \exp \left( { - jk_{0} \sin (\theta_{0} )x_{n} } \right)$$

where $x_{n}$ represents the position information of the recording point on the holographic structure, $k_{r}$ represents the reference wave propagation constant, and $k_{0}$ represents the object wave propagation constant, i.e., the free-space propagation constant. $\theta_{0}$ represents the object wave beam pointing, i.e., the desired beam pointing.

According to the principle of interference, the interference pattern $\psi (x_{n} ,\theta_{0} )$ recorded on the holographic structure can be expressed as:

5 $$\begin{aligned} \psi \left( {x_{n} ,\theta_{0} } \right) & = \left| {\psi_{ref} \left( {x_{n} } \right) + \psi_{obj} \left( {x_{n} ,\theta_{0} } \right)} \right|^{2} \\ & = \left[ {\psi_{ref} \left( {x_{n} } \right) + \psi_{obj} \left( {x_{n} ,\theta_{0} } \right)} \right] \cdot \left[ {\psi_{ref} \left( {x_{n} } \right) + \psi_{obj} \left( {x_{n} ,\theta_{0} } \right)} \right]^{*} \\ & = \psi_{ref}^{2} \left( {x_{n} } \right) + \psi_{obj}^{2} \left( {x_{n} ,\theta_{0} } \right) + \psi_{ref} \left( {x_{n} } \right)\psi_{obj}^{*} \left( {x_{n} ,\theta_{0} } \right) + \psi_{ref}^{*} \left( {x_{n} } \right)\psi_{obj} \left( {x_{n} ,\theta_{0} } \right) \\ \end{aligned}$$

where the interference pattern that contains all the information about the object wave and can be used to reproduce the object wave is the fourth term in Eq. (5), i.e., Eq. (6):

6 $$\psi_{inf} \left( {x_{n} ,\theta_{0} } \right) = \psi_{ref}^{*} \left( {x_{n} } \right)\psi_{obj} \left( {x_{n} ,\theta_{0} } \right) = \exp (j(k_{r} - k_{0} \sin (\theta_{0} ))x_{n} )$$

In the design process of a holographic antenna, it is necessary to determine the holographic patterns corresponding to different beam directions and then construct specific holographic structures according to different holographic patterns. Based on the continuous amplitude weighting method, the holographic structure is constructed by adjusting the amplitude information at $x_{n}$ instead of the phase information. The basic idea of the method is to let the position at $x_{n}$ radiate less energy when the difference between the phase shift value $k_{r} x_{n}$ at $x_{n}$ and the target phase shift value $k_{0} \sin (\theta_{0} )x_{n}$ is large and to let the position at $x_{n}$ radiate more energy when the phase shift value at $x_{n}$ is closer to the target phase shift value. This beam control method of regulating the excitation amplitude can be expressed as a function of amplitude:

7 $$m\left( {x_{n} ,\theta_{0} } \right) = \frac{{\left[ {{\text{Re}} (\psi_{\inf } (x_{n} ,\theta_{0} )) + 1} \right]}}{2} = \frac{{\left[ {\cos [(k_{r} x_{n} - k_{0} \sin (\theta_{0} )x_{n} )] + 1} \right]}}{2}$$

Based on Eq. (7), the contribution rate, denoted as $m\left( {x_{n} ,\theta_{0} } \right)$, of each antenna element to the desired beam direction $\theta_{0}$ can be determined. This contribution rate is a numerical value ranging between 0 and 1, where a higher value indicates a greater contribution of the individual antenna element towards achieving the desired beam direction.

The amplitude excitation value of each unit is discretized as follows: if the value is greater than 0.5, it is considered beneficial for “reproducing” the antenna beam pointing and thus set to “1”; if the value is less than or equal to 0.5, it is deemed not conducive to “reproducing” the antenna beam pointing, and therefore set to “0”. The unit excitation amplitude value can be expressed as:

8 $$I_{onloff} = \left\{ {\begin{array}{*{20}l} 1 \hfill & {m(x_{n} ,\theta_{0} ) > 0.5} \hfill \\ 0 \hfill & {m(x_{n} ,\theta_{0} ) \le 0.5} \hfill \\ \end{array} } \right.\quad (n = 1,2, \ldots ,56)$$

**Figure 4:**
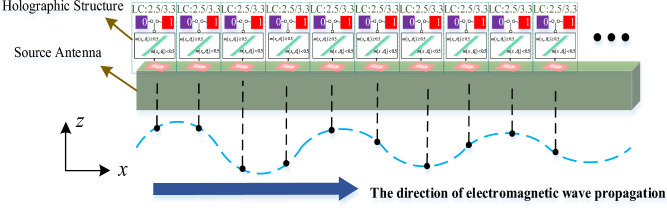
Schematic diagram of holographic antenna radiation.

By utilizing Eq. (8), the binary discretization coding sequence corresponding to each desired beam pointing can be obtained. This is accomplished by modifying the dielectric constant of the LC material to achieve either a "0" or "1" code. Through this method, the antenna can achieve beam scanning functionality. The process and concept of this binary weighted holographic antenna beam steering technique can be illustrated using the holographic antenna radiation diagram presented in Fig. 4.

## Experiments and discussions

### SIW-ML transport structure experiment

**Figure 5:**
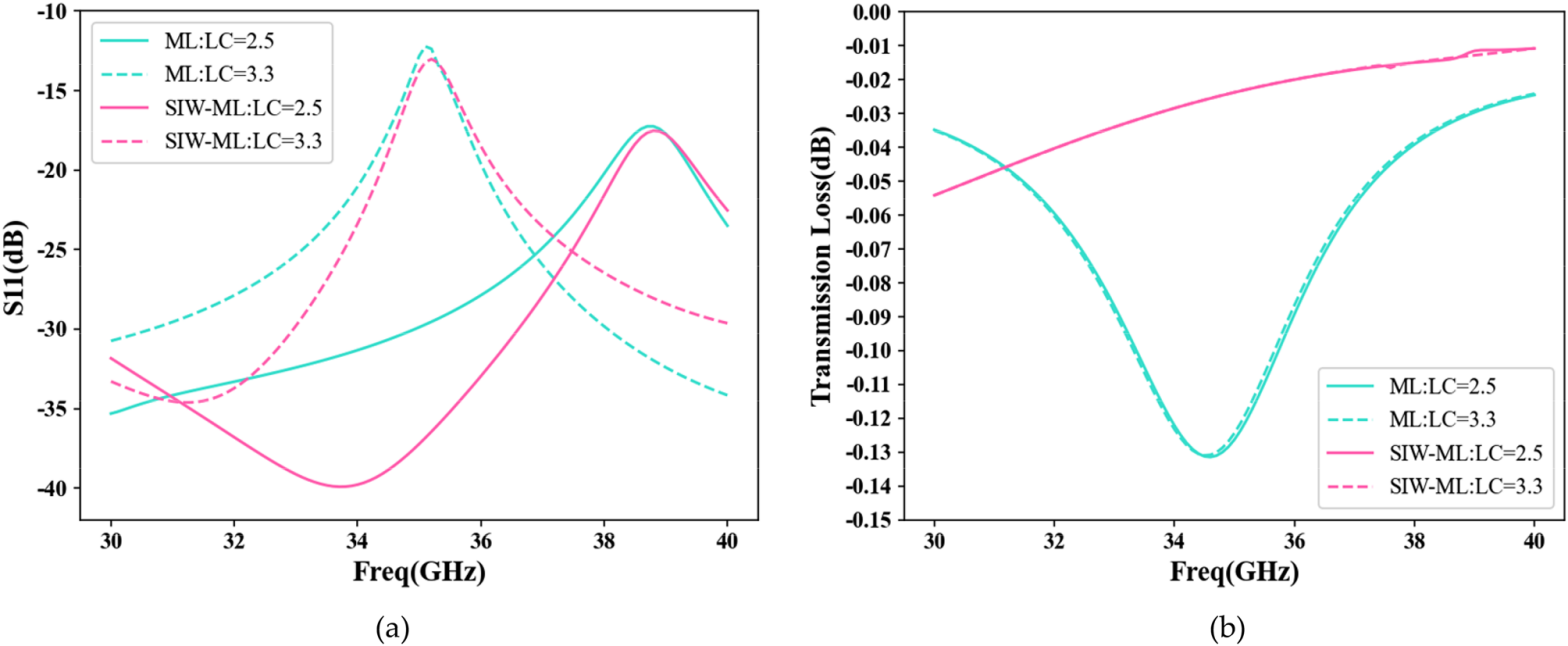
Comparison between ML and SIW-ML. (**a**) S11 comparison; (**b**) Comparison of transmission loss.

The ML transmission structure boasts several advantages, including low cost, easy integration, and high flexibility. However, compared to ML, the SIW is a relatively new transmission structure that provides lower insertion and radiation loss^38,39^. This paper introduces a new SIW-ML transmission structure that combines the SIW structure and the ML structure for the first time. With the addition of two rows of metal columns to the ML transmission medium, inspired by the characteristics of SIW, this novel structure is developed. These metal pillars improve impedance matching and waveguide properties, effectively reducing the total transmission loss, which includes insertion and radiation loss during waveguide transmission (here, transmission loss refers to unfavorable attenuation, not the transmission coefficient S12). The S11 parameters of the two structures are compared in Fig. 5a, revealing that the SIW-ML structure exhibits a lower S11, indicating better impedance matching. When considering only the transmission loss of electromagnetic waves in the transmission medium, without taking into account antenna radiation, the results are shown in Fig. 5b. Changes to the dielectric constant of the LC have little effect on the transmission loss. The SIW-ML structure demonstrates significantly lower transmission loss than the ML structure over the frequency range of 30–40 GHz. This suggests that the SIW-ML structure can reduce unnecessary attenuation of electromagnetic waves and improve energy utilization efficiency.

**Figure 6:**
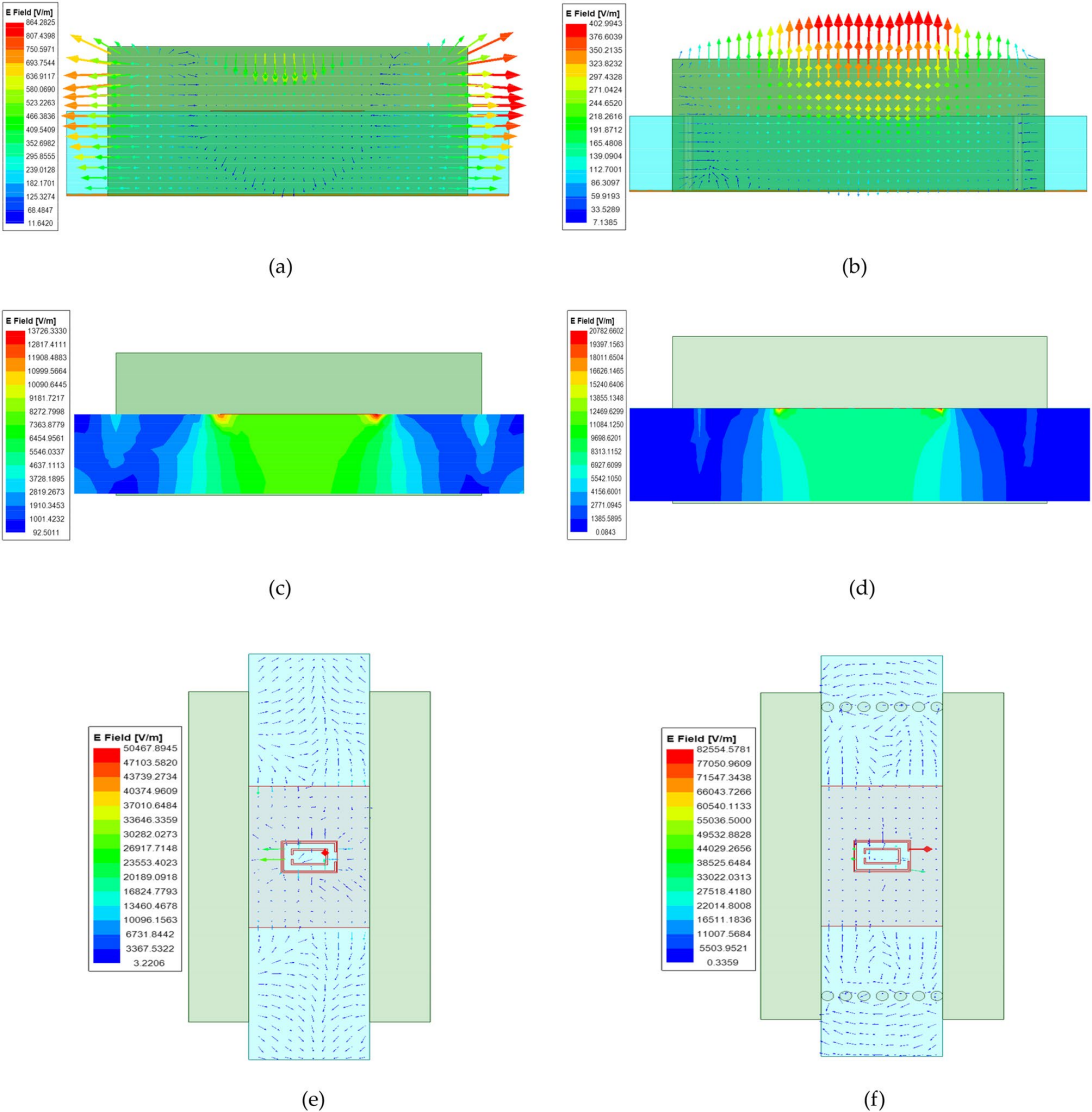
Comparison of ML and SIW-ML field distributions. (**a**) Electric field vector of input wave port of ML structure; (**b**) Electric field vector of input wave port of SIW-ML structure; (**c**) Field energy distribution of output wave port of ML structure; (**d**) Field energy distribution of output wave port of SIW-ML structure; (**e**) Vector distribution of electric field of ML structure; (**f**) Vector distribution of electric field of SIW-ML structure.

The interaction between the metal pillar and the transmission medium in the SIW-ML structure enhances the energy coupling effect, leading to improved signal transmission efficiency. Through careful design of the shape and position of the metal pillar, the propagation path of the electromagnetic wave can be controlled, allowing for more concentrated signal transmission to the desired area and reducing beam diffusion. Figure 6a–d depict the electric field vector diagram at the input wave port and the electric field energy diagram at the output wave port for both ML and SIW-ML structures. It is evident that the SIW-ML structure enables more intensive transmission of signal energy in the central channel, resulting in reduced beam diffusion and higher electric field energy values. Additionally, Fig. 6e and f demonstrate that the SIW-ML structure effectively controls the transmission of electromagnetic waves, with significantly larger peak electric field values compared to the ML structure. Moreover, the substrate can serve as a structural support, providing mechanical stability and protecting the antenna.

### Antenna unit experiment

**Figure 7:**
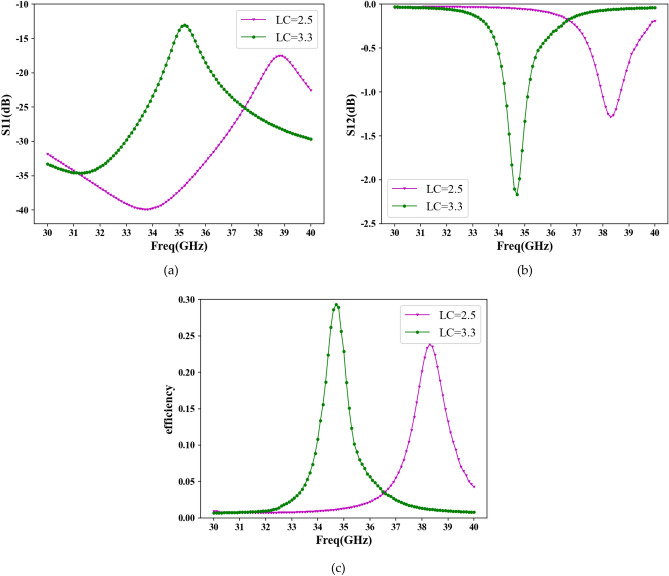
Antenna unit simulation results. (**a**) S11 parameter; (**b**) S12parameter; (**c**) radiation efficiency.

The design of the antenna unit plays a critical role in constructing of a periodic leaky-wave antenna. The antenna unit should not only have excellent impedance matching performance but also have appropriate radiation efficiency. Excessive radiation efficiency causes guided waves propagating through a few units in the medium to be radiated entirely, thereby failing to accomplish periodic antennas. Conversely, low radiation efficiency results in a low antenna gain. We use HFSS electromagnetic simulation software to simulate the antenna unit. Figure 7 shows the S11 parameter, S12 parameter and the radiation efficiency of the antenna unit.

The simulation results for the antenna unit, including S11, S12, and radiation efficiency, are depicted in Fig. 7. It can be observed that the S11 parameters remain below − 13 dB throughout the frequency range, indicating excellent impedance matching performance of the antenna. The resonant frequency of the antenna unit is determined by analyzing the S12 parameters and radiation efficiency, and it changes as the dielectric constant of the LC material is varied between 2.5 and 3.3. Additionally, it is evident that the resonant characteristics of the antenna improve with the incorporation of the CSRR structure. At a dielectric constant of 3.3, the resonant frequency measures 34.7 GHz, and the antenna unit demonstrates the highest radiation efficiency when equipped with the LC material at this dielectric constant. However, with a dielectric constant of 2.5, the antenna unit exhibits minimal energy radiation, with a radiation efficiency below 2%. This characteristic renders it suitable for subsequent designs of periodic leaky-wave antennas.

### Single-beam experiment with periodic leaky-wave antenna

#### One-dimensional array antenna structure

**Figure 8:**
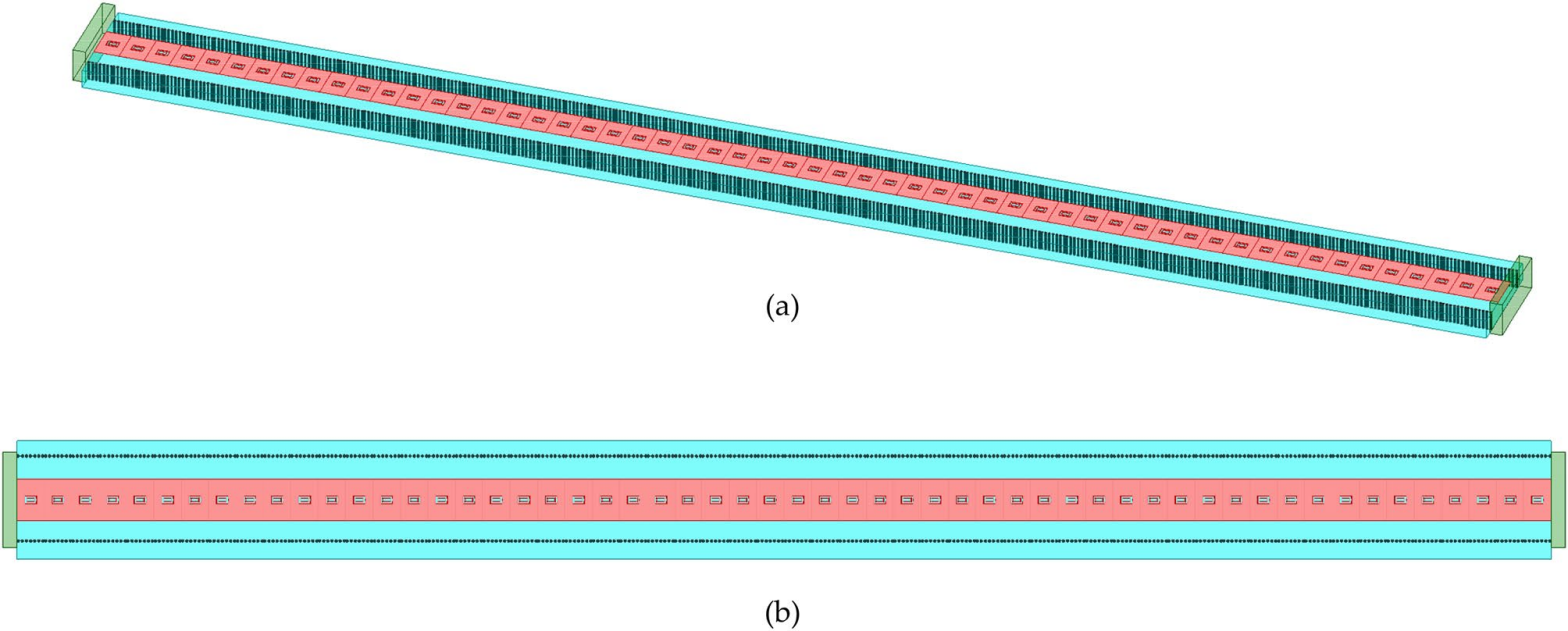
The structure of periodic leaky-wave antenna. (**a**) Main view; (**b**) top view.

Arranging 56 antenna units in a one-dimensional manner, the spacing between units has a significant impact on the radiation characteristics of the antenna. When the unit spacing is $\lambda /2$, there will be significant reflection between adjacent units, leading to abnormal transmission of electromagnetic waves. When the unit spacing exceeds $\lambda /2$, according to array antenna theory, grating lobes appear within the visible region of the array antenna, resulting in a decrease in gain. While a smaller cell spacing can lead to a more accurate recording of the holographic pattern in the structure, it also produces a stronger mutual coupling effect, which can deteriorate the antenna pattern and increase the difficulty of analysis. Taking these factors into account, a spacing of 2mm is utilized between the units. Thus, the total length of the one-dimensional antenna array in this design is 112mm. A schematic diagram illustrating the structure of the one-dimensional antenna array is presented in Fig. 8.

#### Single-beam experiment

At the operating frequency of 34.7 GHz, Fig. 7c demonstrates that the radiation efficiency is higher when the dielectric constant of the LC material is set to 3.3. To achieve beam scanning functionality, binary discrete amplitude weighting is employed, and the corresponding binary discrete coding sequence for each desired beam pointing can be obtained using Eq. (8). By modifying the dielectric constant of the LC material, either a “0” or “1” code can be achieved. Table 3 presents the binary discrete amplitude-weighted sequences of 56 cells corresponding to different beam pointings. In this table, “1” indicates that the dielectric constant of the cell's LC material is set to 3.3, while “0” indicates that the dielectric constant of the cell's LC material is set to 2.5.

**Table 3:** The binary discrete amplitude-weighted sequences of 56 units corresponding to different beam pointings.

Directions	Binary discrete amplitude-weighted sequences
0°	0, 1, 0, 1, 0, 1, 0, 1, 0, 1, 0, 1, 0, 1, 0, 1, 0, 1, 0, 1, 0, 1, 1, 0, 1, 0, 1, 0, 1, 0, 1, 0, 1, 0, 1, 0, 1, 0, 1, 0, 1, 0, 1, 1, 0, 1, 0, 1, 0, 1, 0, 1, 0, 1, 0, 1
− 30°	0, 1, 0, 1, 1, 0, 1, 1, 0, 1, 0, 0, 1, 0, 0, 1, 0, 1, 1, 0, 1, 0, 0, 1, 0, 0, 1, 0, 1, 1, 0, 1, 1, 0, 1, 0, 0, 1, 0, 1, 1, 0, 1, 1, 0, 1, 0, 0, 1, 0, 0, 1, 0, 1, 1, 0
30°	1, 0, 1, 0, 1, 1, 0, 1, 0, 1, 0, 0, 1, 0, 1, 0, 0, 1, 0, 1, 0, 1, 1, 0, 1, 0, 1, 1, 0, 1, 0, 1, 1, 0, 1, 0, 1, 0, 0, 1, 0, 1, 0, 0, 1, 0, 1, 0, 1, 1, 0, 1, 0, 1, 1, 0
− 53°	0, 1, 1, 0, 1, 1, 0, 0, 1, 0, 0, 1, 1, 0, 0, 1, 0, 0, 1, 1, 0, 1, 1, 0, 0, 1, 1, 0, 1, 1, 0, 0, 1, 0, 0, 1, 1, 0, 1, 1, 0, 0, 1, 1, 0, 1, 1, 0, 0, 1, 0, 0, 1, 1, 0, 0
− 60°	1, 0, 1, 1, 0, 1, 1, 0, 0, 1, 0, 0, 1, 0, 0, 1, 0, 0, 1, 0, 0, 1, 0, 0, 1, 1, 0, 1, 1, 0, 1, 1, 0, 1, 1, 0, 1, 1, 0, 0, 1, 0, 0, 1, 0, 0, 1, 0, 0, 1, 0, 0, 1, 0, 0, 1

**Figure 9:**
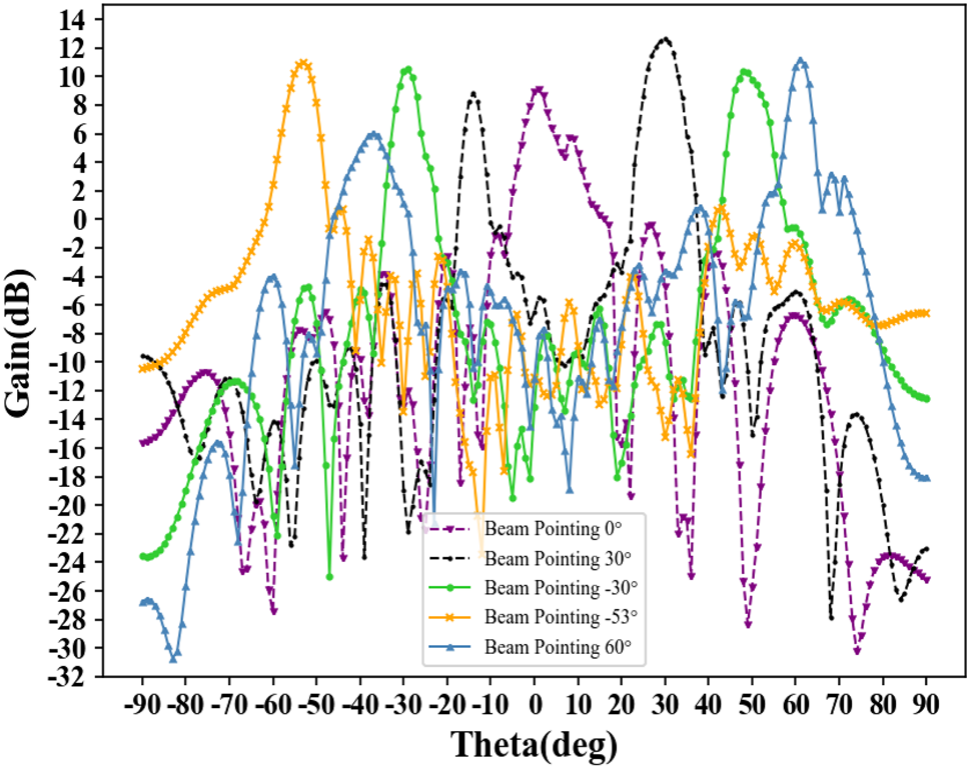
Different beam pointing plots based on binary discrete amplitude weighting (at 34.7GHz).

Figure 9 shows the single-beam pointing diagrams corresponding to the discrete sequences in Table 2 when operating at 34.7 GHz.

**Figure 10:**
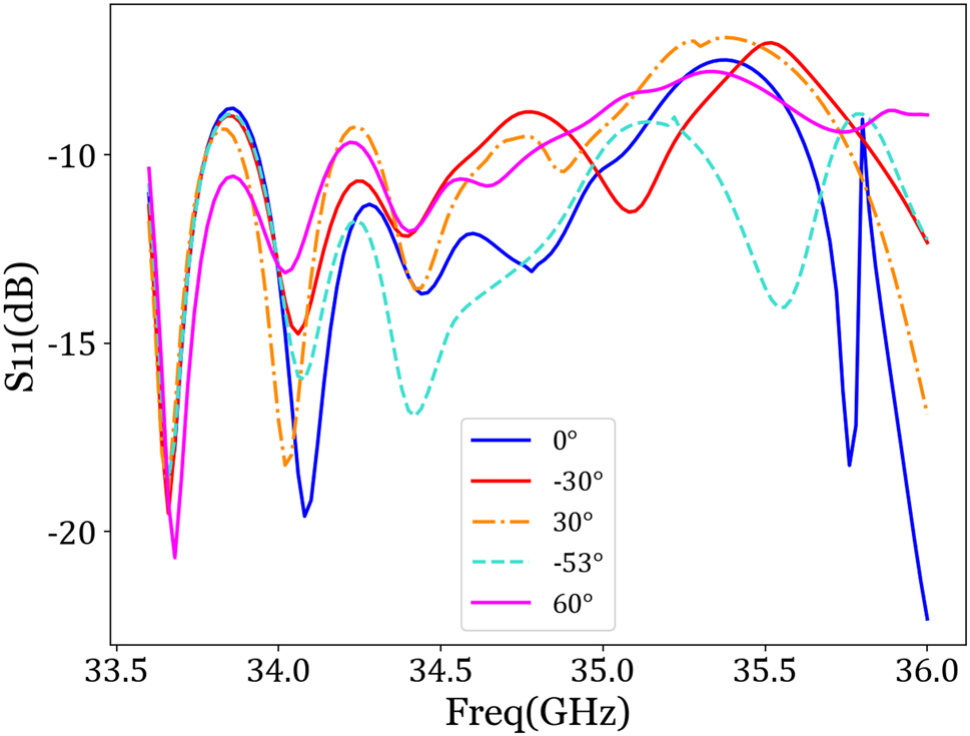
Simulation of antenna reflection coefficient.

Figure 10 shows that the reflection coefficient (i.e., the S11 parameter) is essentially below − 10 dB in the 34–35 GHz frequency range over the beam sweep from − 53° to + 60°, indicating a good impedance match.

From Fig. 9, it can be observed that the different beam pointings are consistent with expected values, with gains mostly above 10 dB. Especially at + 30°, with an angular error of 1°, the gain reaches 12.6342 dB. Through simulation experiments, scanning angles ranging from − 53° to + 60° are achieved at 34.7 GHz. Table 4 lists the specific angular errors and main beam gains at different beam pointings.

**Table 4:** Angular errors and gains at different beam pointings.

Preset angles	− 53°	− 30°	0°	+ 30°	+ 60°
Actual angles	− 53.9°	− 31.1°	+ 0.7°	+ 31°	+ 62.2°
Angular error	0.9°	1.1°	0.7°	1°	2.2°
Gain (dB)	10.9943 dB	10.3324 dB	9.1257 dB	12.6342 dB	11.2192 dB

**Figure 11:**
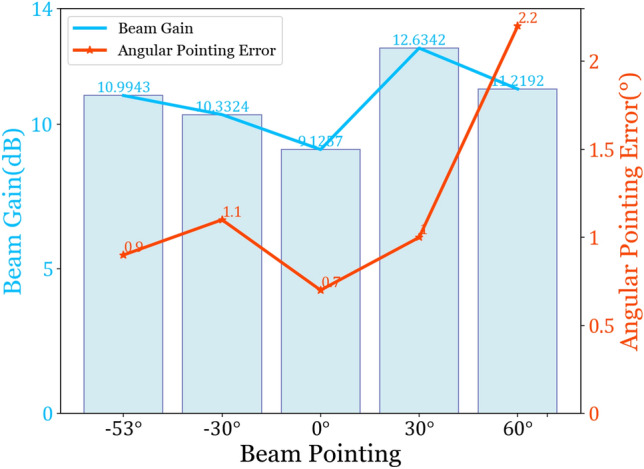
Beam gain and angle error.

According to Table 4, we can conclude that the beam pointing error of the 1D array antenna ranges from 0.7° to 2.2°. The beam gain falls between 9.1257 and 12.6342 dB, with a fluctuation range of approximately 3 dB. For a clearer analysis of the gain and deviation of each beam, Fig. 11 presents a comparative graph.

Furthermore, Fig. 9 reveals that while some beam pointings align with the expected values, there are also sub-beams in other directions. For example, when the expected direction is − 30°, there is a sub-beam at + 50°; when the expected direction is + 30°, there is a sub-beam at − 20°. This occurs because the similarity error in the angular pointing arises due to the use of discrete amplitude weighting. When there is a certain degree of similarity between the two pointing coding sequences, similar situations may occur, but it does not affect the main beam pointing. This situation can be solved by reducing the unit spacing and increasing the number of units.

### Multi-beam experiment with periodic leaky-wave antenna

Multi-beam antennas have the capability to generate multiple high-gain beams within a specific area, thereby offering wider coverage and significantly enhancing communication capacity and spectral efficiency. These antennas play a critical role in various domains such as radar, satellite communication, and wireless communication. By leveraging the beam algorithm rooted in the holographic principle, not only can control over a single beam be achieved, but it also enables the realization of more intricate multi-beam radiation patterns. When there are multiple expected angles, modifications to the objective wave function and amplitude function in Eqs. (4) and (7) are required. The objective wave function and amplitude function of multi-beam can be expressed as:

9 $$\psi_{obj} \left( {x_{n} ,\theta_{0} \ldots \theta_{m} } \right) = \exp ( - jk_{0} \sin \theta_{0} x_{n} ) + \exp ( - jk_{0} \sin \theta_{1} x_{n} ) + \cdots \cdots + \exp ( - jk_{0} \sin \theta_{m} x_{n} )$$

10 $$m\left( {x_{n} ,\theta_{0} ,\theta_{1} ,...,\theta_{m} } \right) = \left\{ {\frac{{\sum\nolimits_{m = 0}^{M - 1} {\frac{{\left[ {\cos [(k_{r} x_{n} - k_{0} \sin (\theta_{m} )x_{n} )] + 1} \right]}}{2}} }}{M}} \right\}$$

The continuous amplitude in Eq. (10) is discretized in two values to obtain as shown in Eq. (11):

11 $$I_{onloff} = \left\{ {\begin{array}{*{20}l} 1 \hfill & {m(x_{n} ,\theta_{0} ,\theta_{1} , \ldots ,\theta_{m} ) > 0.5} \hfill \\ 0 \hfill & {m(x_{n} ,\theta_{0} ,\theta_{1} , \ldots ,\theta_{m} ) \le 0.5} \hfill \\ \end{array} } \right.\quad (n = 1,2, \ldots ,56)$$

At 34.7 GHz, a dual-beam holographic metasurface leaky-wave antenna based on LC is designed. The initial angles are set to − 30° and + 30°, − 10° and + 10° and − 30°, + 50° and + 10°, 0° and + 30°, respectively, and substituted into Eqs. (10) and (11). The dielectric constant of each uni's LC material is set according to the calculated binary discrete amplitude-weighted value. Table 5 lists the binary discrete amplitude-weighted sequences of the 56 units for different multi-beam pointings. In this table, “1” indicates that the dielectric constant of the corresponding unit's LC material is set to 3.3, while “0” indicates a dielectric constant of 2.5.

**Table 5:** Binary discrete amplitude-weighted sequences of 56 units corresponding to different multi-beam pointings.

Directions	Binary discrete amplitude-weighted sequences
0°/+ 30°	1, 0, 1, 0, 0, 1, 0, 1, 0, 1, 0, 1, 0, 0, 0, 1, 0, 1, 0, 1, 0, 1, 1, 0, 1, 0, 1, 0, 1, 1, 1, 0, 1, 0, 1, 0, 1, 0, 1, 1, 0, 1, 0, 0, 1, 0, 1, 0, 0, 1, 0, 1, 0, 1, 0, 1
− 10°/+ 10°	0, 1, 0, 1, 0, 1, 1, 0, 1, 0, 1, 0, 1, 0, 1, 0, 1, 0, 1, 1, 0, 1, 1, 0, 1, 0, 1, 0, 1, 0, 1, 1, 0, 1, 0, 1, 0, 1, 0, 1, 0, 1, 0, 0, 0, 1, 0, 1, 0, 1, 0, 1, 0, 1, 0, 1
− 30°/+ 30°	0, 1, 1, 0, 1, 0, 0, 1, 0, 1, 0, 0, 1, 0, 1, 1, 0, 1, 0, 0, 1, 0, 0, 1, 1, 0, 1, 0, 0, 1, 0, 1, 1, 0, 1, 0, 1, 1, 0, 1, 0, 0, 1, 1, 0, 0, 1, 0, 1, 0, 0, 1, 0, 1, 1, 0
− 30°/+ 50°	0, 1, 0, 1, 1, 0, 0, 1, 0, 1, 0, 1, 1, 1, 0, 1, 0, 1, 0, 0, 1, 0, 0, 1, 0, 0, 1, 0, 1, 0, 0, 0, 1, 0, 1, 0, 1, 1, 0, 0, 1, 0, 1, 1, 0, 1, 0, 1, 1, 1, 0, 1, 0, 1, 0, 1

**Figure 12:**
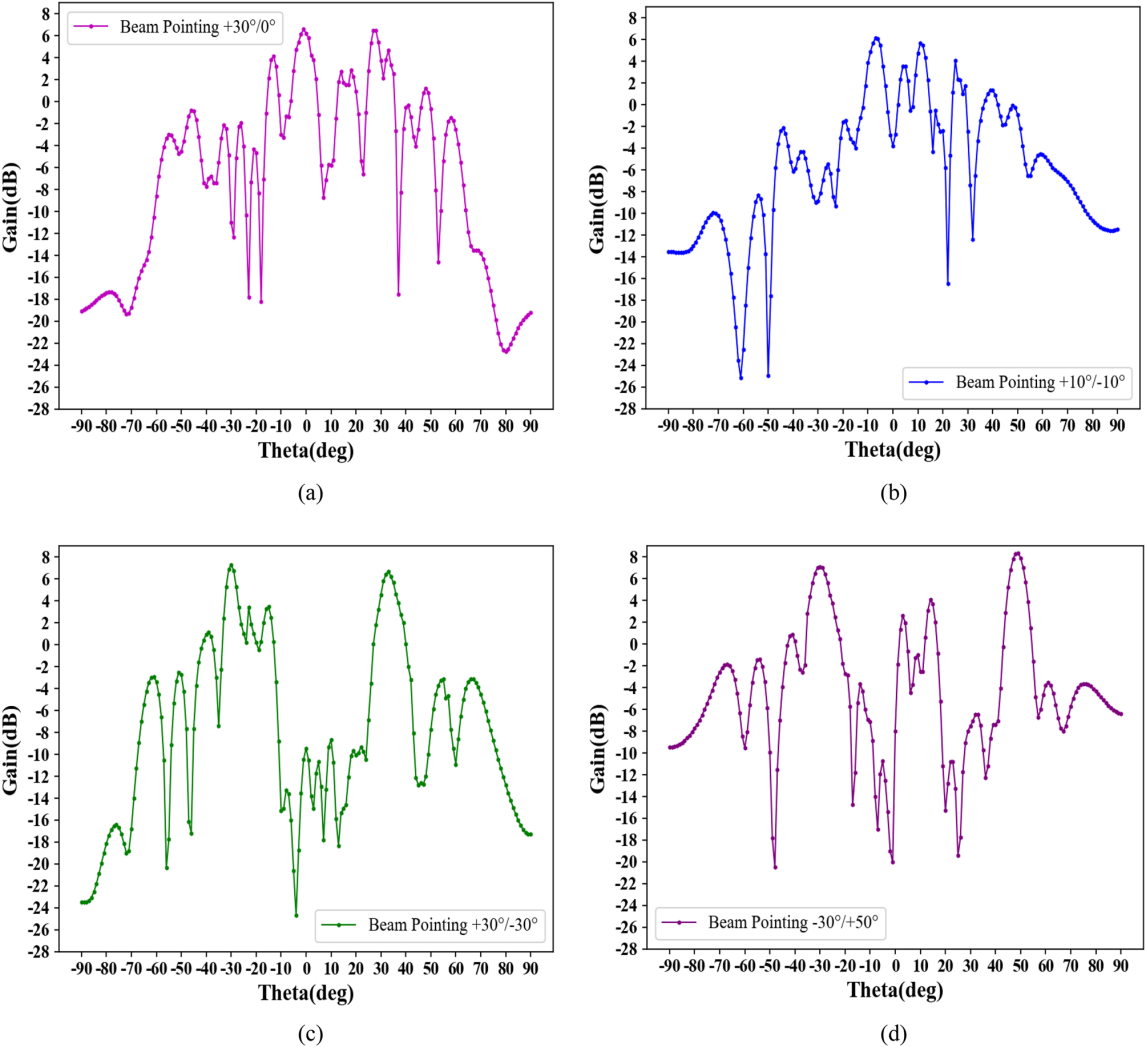
Multi-beam radiation plots (at 37.4 GHz) (**a**) + 30°and0°; (**b**) − 10°and + 10°; (**c**) − 30° and + 30°; (**d**) − 30° and + 50°.

Figure 12 illustrates the multi-beam pointing diagrams corresponding to the discrete sequences in Table 4, when operating at 34.7 GHz.

In Fig. 12a, it can be observed that the actual angles are − 1.4° and + 27.9°, with a maximum angular error of 2.1°. Figure 12b shows that the actual angles are − 8.3° and + 11.8°, with a maximum angular error of 1.8°. Moving to Fig. 12c, the actual angles are − 31.5° and + 33.1°, with a maximum angular error of 1.5°. Finally, in Fig. 12d, the actual angles are − 30.6° and + 48.9°, with a maximum angular error of 1.1°. These findings suggest that the utilization of discrete amplitude-weighted techniques for multi-beam scanning leads to greater angular errors than single-beam scanning. Nevertheless, these errors fall within an acceptable range, providing evidence of the efficacy of binary control to meet multi-beam design requirements.

## Conclusion

In recent years, there has been an increasing demand for high-frequency bands in millimeter-wave communication for 5G. As a result, research on leaky-wave antennas needs to focus on higher frequency bands. Currently, most of the research on leaky-wave antennas is conducted in the frequency band below 30 GHz, with limited studies on the Ka-band above 30 GHz. Existing research results have highlighted several issues, including poor impedance matching performance, low antenna gain, large antenna volume, weak resonance, and limited beam scanning range. Responding to these issues, this paper proposes an ML antenna unit based on LC etched CSRR. Additionally, the SIW-ML transmission structure is proposed in a first-of-its-kind approach by combining the SIW structure with the ML transmission structure. This approach enhances signal transmission efficiency compared to ML, reducing insertion loss and radiated loss in waveguide transmission while improving antenna bandwidth performance. The proposed design also helps reduce beam spread in leaky-wave antennas, allowing for more concentrated signal transmission to the target area. This reduces the degree of beam diffusion and improves impedance matching. Moreover, the SIW-ML structure serves as a mechanical support, ensuring the antenna's mechanical stability and protection. Experimental results demonstrate that the antenna exhibits excellent resonance characteristics at 34.7 GHz, with S11 parameters below − 13 dB within the frequency range of 30–40 GHz, indicating good impedance matching performance. By arranging 56 antenna elements to form a one-dimensional periodic leaky-wave antenna array, the antenna achieves an angle scan between − 53° and + 60° at 34.7 GHz, with a gain of 12.63 dB. After successfully implementing single-beam scanning, the paper presents a method that combines LC materials, discrete amplitude weighting technology, and multi-beam theory to enable multi-beam scanning. Experiments show that the designed 56-element antenna can well realize beam scanning in both directions.

In future work, a 2D leaky-wave antenna array will be further designed based on the antenna unit designed in this paper. It can accomplish a wide angular scanning range and high gain in 2D space.

## Data Availability

The data in this manuscript is available upon reasonable request from the corresponding authors.
